# Hydrolysis of Virgin Coconut Oil Using Immobilized Lipase in a Batch Reactor

**DOI:** 10.1155/2012/542589

**Published:** 2012-08-16

**Authors:** Lee Suan Chua, Meisam Alitabarimansor, Chew Tin Lee, Ramli Mat

**Affiliations:** ^1^Metabolites Profiling Laboratory, Institute of Bioproduct Development, Universiti Teknologi Malaysia, Johor, 81310 Johor Bahru, Malaysia; ^2^Department of Chemical Engineering, Faculty of Chemical Engineering, Universiti Teknologi Malaysia, Johor, 81310 Johor Bahru, Malaysia; ^3^Deparment of Bioprocess Engineering, Faculty of Chemical Engineering, Universiti Teknologi Malaysia, Johor, 81310 Johor Bahru, Malaysia

## Abstract

Hydrolysis of virgin coconut oil (VCO) had been carried out by using an immobilised lipase from *Mucor miehei* (Lipozyme) in a water-jacketed batch reactor. The kinetic of the hydrolysis was investigated by varying the parameters such as VCO concentration, enzyme loading, water content, and reaction temperature. It was found that VCO exhibited substrate inhibition at the concentration more than 40% (v/v). Lipozyme also achieved the highest production of free fatty acids, 4.56 mM at 1% (w/v) of enzyme loading. The optimum water content for VCO hydrolysis was 7% (v/v). A relatively high content of water was required because water was one of the reactants in the hydrolysis. The progress curve and the temperature profile of the enzymatic hydrolysis also showed that Lipozyme could be used for free fatty acid production at the temperature up to 50°C. However, the highest initial reaction rate and the highest yield of free fatty acid production were at 45 and 40°C, respectively. A 100 hours of initial reaction time has to be compensated in order to obtain the highest yield of free fatty acid production at 40°C.

## 1. Introduction

Coconut oil, which is derived from the seeds of coconut palm, *Cocos nucifera, *is traditionally processed from the meat of the fruit, called copra. Copra is the dried kernel that produced by smoke drying, sun drying, or a combination of both methods. Therefore, it is usually colorless to pale brownish yellow. 

Recently, the most welcomed product from coconut is virgin coconut oil (VCO), particularly from the tropical countries. The concept of producing VCO is actually triggered by the well-known virgin olive oil that produced from Mediterranean Basin. The high demand for the virgin oils is definitely due to the preservation of oil composition, including the minor components such as provitamin A, vitamin E, phytosterols, and polyphenols, without aflatoxin contamination and oxidative rancidity from drastic processing and handling approach. These minor components are believed to have the nutritional benefits. By definition, VCO is defined as the oil obtained from the fresh, mature kernel of coconuts by mechanical or natural means without the use of heat, chemical refining, bleaching, and odorizing which does not lead to the alteration of the natural content of the oil [1]. It should also have the moisture content less than 0.1%. 

Because of the beneficial effects on human health [2, 3] and high saturation degree [1] as well as high oxidative stability of VCO [4], the oil is the great source of oil material for the production of value-added structural fats and oils. Furthermore, the pleasant odor and taste of coconut oil may enhance the quality of the fat blend. Hydrogenated fats that are commonly used in confectionary products always associated with coronary diseases [5] due to the presence of significant level of *trans*fatty acids (2–13%). Hence, it is crucial to encourage studies on producing healthier fat/oil stocks from VCO. The production of the modified fats/oils might require a series of reaction processes such as hydrolysis, interesterification, and transesterification dependant on the end-product application. 

The lipid composition [6, 7] and the physicochemical properties [8, 9] of VCO have been reported. However, the scientific data on VCO hydrolysis and its kinetic study is very limited in addition to the reaction optimization. In the present study, hydrolysis of VCO was carried out by using immobilized lipase in a well-stirred batch reactor. This enzymatic fat-splitting reaction produces free fatty acids and glycerols with fewer undesirable byproducts formation because of mild reaction conditions. To our knowledge, the natural fatty acids produced from the natural techniques are more preferable, especially in nutraceutical, cosmeceutical, and pharmaceutical industries. The dominant fatty acids, medium-chain fatty acids are mainly used as nutritional supplement and in formulation of infant food. The use of immobilized lipase offers many advantages such as enzyme reusability, high stability of enzyme, less downstream process and predictable production yield. 

## 2. Materials and Methods 

### 2.1. Chemicals and Materials

An 1,3-specific immobilized lipase (Lipozyme) from *Mucor miehei* was purchased from Sigma-Aldrich (USA). VCO samples were provided by Institute of Bioproduct Development, Universiti Teknologi Malaysia, Malaysia. The chemicals and solvent, sodium hydroxide, tributyrin, and n-hexane, were purchased from Fluka Chemie AG, Switzerland.

### 2.2. Enzyme Assay

The activity of the immobilised lipase was assayed using tributyrin as the substrate in a water-jacketed vessel [10]. A 20 mL of tributyrin and 2 mL water were added into n-hexane to make up the total reaction solution to 50 mL. The solution was stirred at 300 rpm and maintained at 40°C. The reaction was initiated by adding 0.5 g of the immobilized lipase into the solution. Samples (0.5 mL) were withdrawn for sodium hydroxide (0.09 M) titration until the reaction reached equilibrium. The amount of sodium hydroxide (0.09 M) added is proportionally equal to the amount of butyric acid liberated from hydrolysis. The activity of Lipozyme from *Mucor miehei* was found to be 189.0 U/g. This enzyme activity was expressed in lipase unit (LU), where 1 LU is defined as the amount of enzyme required to liberate 1 mmole of butyric acid per minute.

### 2.3. Hydrolysis of VCO

VCO was hydrolyzed by Lipozyme in a well-stirred batch reactor. The optimum conditions for the reaction were determined by varying the parameters such as substrate concentration, enzyme loading, temperature, and water content. For each experiment, an appropriate amount of n-hexane was added to the total volume of 50 mL. Samples (0.5 mL) were taken at interval time until the reaction reached equilibrium. All experiments were carried out in triplicate, and the mean value was reported with the standard deviation of less than 0.05 mmol/min.

### 2.4. Varying Reaction Parameters

The effect of substrate concentration (VCO) was studied by varying the volume of VCO from 10 to 48 mL in the reaction mixture, while fixing the other parameters as follows: enzyme loading, 0.5 g; water content, 2 mL; reaction temperature, 40°C. When 2 mL of water was added to the 48 mL of VCO, the reaction mixture was in solvent-free system. 

The water content was varied from 1 to 5 mL (2 to 10% (v/v)) at the fixed conditions as follows: substrate concentration, 0.4% (v/v); enzyme loading, 0.5 g; reaction temperature, 40°C.

To optimize the reaction temperature, the temperature was varied from 30 to 50°C. The other parameters were fixed as above, unless otherwise stated. 

The optimum loading for enzyme was also determined by varying the enzyme quantity from 0.2 to 0.6 g.

### 2.5. Sample Analysis

The progress of the reactions was monitored by using titration method. Sodium hydroxide (0.09 M) was used as the titrant with phenolphthalein as indicator. The preference of titration method is mainly because it requires lesser time compared to gas chromatography technique. The content of free fatty acids liberated from hydrolysis could be determined within 1 min of time. Furthermore, the results obtained by titration and gas chromatography approach have been reported about 10% of difference, which was relatively low [9]. 

For confirmation, the free fatty acid content was analyzed by gas chromatography integrated with a flame ionization detector (Shimadzu GC-17A, Kyoto, Japan). A Nukol column (Supelco, USA) with the dimension of 0.5 *μ*m × 0.53 mm × 15 m was used for separation with a flow rate of 104 mL/min at 100 kPa. The major fatty acids: caproic acid (C6), capric acid (C10), and lauric acid (C12) which had been identified from the triglyceride composition of VCO [8] were used as the standard chemicals to determine the free fatty acid content. Samples were diluted with n-hexane prior to GC injection. The temperatures of injector and detector were set at 220°C. The column temperature was programmed to rise from 110 to 220°C at the increase rate of 8°C per minute. The injection volume was 1 *μ*L. 

## 3. Results and Discussion

### 3.1. Effect of Enzyme Concentration

**Figure 1:**
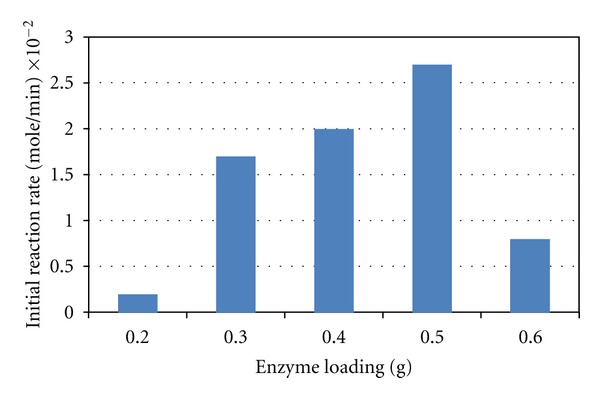
Initial reaction rates of free fatty acid liberation from VCO hydrolysis at different amounts of enzyme loading.

**Figure 2:**
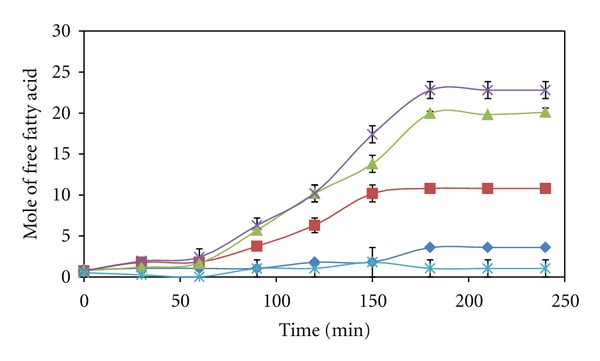
Reaction progress curves of VCO hydrolysis catalyzed by Lipozyme at different amounts of enzyme loadings from 0.2 (♦), 0.3 (■), 0.4 (▲), 0.5 (×) to 0.6 g (∗).

In an ideal condition, an increase of enzyme concentration would proportionally increase the reaction rate. However, this proportional relationship was not observed in the VCO hydrolysis catalyzed by lipase loaded from 0.2 to 0.6 g at the fixed reaction conditions. The increase of enzyme loading increased the reaction rate up to a critical value at 0.5 g which is equal to 1% (w/v) as shown in Figure 1. Beyond this value, the further increase of enzyme loading did not increase, but reduced the hydrolysis rate significantly. The reduction in the hydrolysis rate has also significantly reduced the amount of free fatty acids liberated at the equilibrium condition (Figure 2). This could be explained by the limitation of interfacial area for catalysis. The limitation was caused by the saturation of enzymes in the bulk phase, hence reducing the flexibility of enzyme during catalysis. This phenomenon of interfacial area limitation has also been reported in the study of palm oil hydrolysis catalyzed by lipases from *Candida rugosa* [11]. 

### 3.2. Effect of VCO Concentration

**Figure 3:**
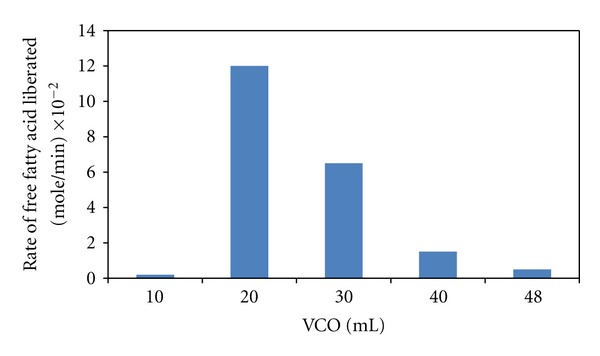
Initial reaction rates of free fatty acid liberation from VCO hydrolysis at different volumes of VCO.

The concentration of VCO influenced the initial reaction rate and the yield of free fatty acid liberated from hydrolysis. The initial reaction rate was increased sharply from 20 to 40% (v/v) of VCO concentration and then decreased gradually from 60 to 96% (v/v) as presented in Figure 3. The solvent-free system has the lowest initial reaction rate, namely, at the VCO concentration of 96% (v/v). This observation explains the phenomenon of substrate inhibition. Substrate inhibition has been reported in the lipase-catalyzed hydrolysis of edible oils, but not for VCO. In fact, substrate inhibition would occur at different substrate concentration dependent on the type of enzyme, the nature of oil, and the reaction conditions. Al-Zuhair et al. [11] reported that substrate inhibition was observed in the palm oil hydrolysis at the concentration above 30–40% (v/v). Their result was in line with the finding of this study, where substrate inhibition due to the high VCO content was observed at the concentration more than 40% (v/v). The close findings of substrate inhibition concentration between palm oil and virgin coconut oil might be because of the high content of short- to medium-chain saturated fatty acids from the palm tree family. 

**Figure 4:**
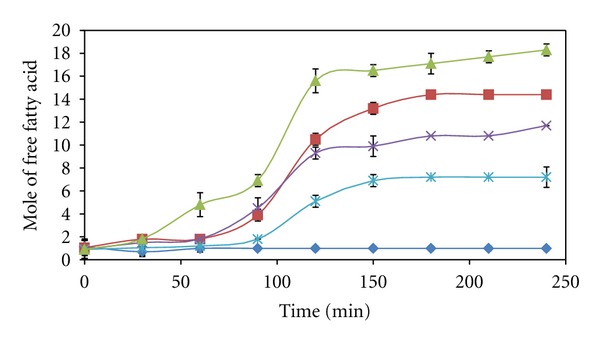
Reaction progress curves of VCO hydrolysis catalyzed by Lipozyme at different VCO concentrations from 20 (♦), 40 (■), 60 (▲), 80 (×) to 96% (v/v) (∗).

Even though the initial reaction rate of VCO hydrolysis was the highest at the concentration of 40% (v/v), and the yield of free fatty acid liberated from the reaction was about 27% lower than the yield achieved at the VCO concentration 60% (v/v) as shown in Figure 4. The enzyme active sites might be saturated by VCO, which would reduce the diffusion rate of substrate or product in/out from the active sites. The further increase of VCO concentration reduced the accessibility of enzyme active sites significantly. 

The kinetic studies of the reaction were carried out using the approach of initial rate analysis. The maximum velocity of lipase (**V*_max⁡_*) and its Michaelis constant (*K*_*m*_) were estimated from the Michaelis-Menten equation using the plot of Hanes-Woolf for linearization. The **V*_max⁡_* and *K*_*m*_ values of Lipozyme in the hydrolysis of VCO were 160 mM/min and 42.42% (v/v), respectively. 

### 3.3. Effect of Water Content

The water content is one of the most important parameters to be investigated, especially in the enzymatic reaction in organic media. Water reacts as reactant in the hydrolysis and the modifier for lipase functionality during reaction [12]. The amount of water present in the system will affect the reversibility of reaction either toward hydrolysis or esterification direction. 

**Figure 5:**
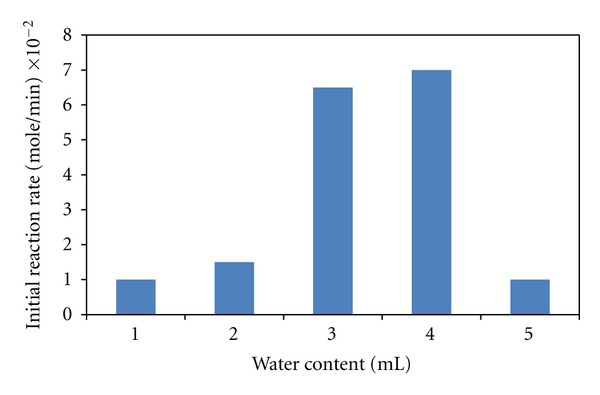
A bell-shape curve of initial reaction rate of VCO hydrolysis versus the content of water added into the reaction media.

The water content of VCO hydrolysis was varied from 1 to 5 mL (2 to 10% (v/v)) in this study (Figure 5). The initial rate of hydrolysis showed a bell-shape curve at different amount of water added into the reaction media. Referring to Figure 5, the optimum water content was about 3.5 mL or equal to 7% (v/v). This value was about twofold higher than the critical water content at 3.6% (v/v) for the hydrolysis of short chain ester, namely, tetrahydrofurfuryl butyrate [12]. 

Since lipases are the interfacial enzymes, the presence of water in excess will cause the water layer around the enzyme surface become thicker. The thickness of the water layer has significant effect on the diffusivity of substrate and product from the enzyme active sites. The low solubility of VCO and fatty acids in aqueous medium has caused the problem of diffusion and low reaction rate. At the water content higher than 6% (v/v), the enzyme particles started to aggregate and stick to the surface of glass reactor because of surface tension effect. The similar observation has been reported by Chua and Sarmidi [13] in their experiments using immobilized lipases in organic media. 

Too excessive water content in the reaction media might denature the protein content of enzyme particles permanently. Therefore, immobilized lipases usually have higher resistance toward denaturation contributed by high water content than free enzymes. The aim of immobilization is to maintain the three-dimensional active form of enzymes and to have higher resistance toward extreme reaction conditions. 

### 3.4. Effect of Temperature

**Figure 6:**
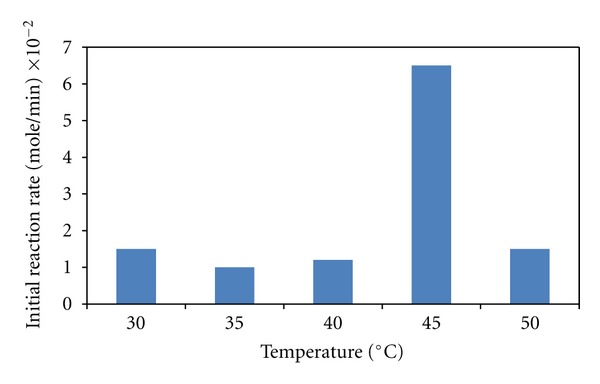
Initial reaction rates of free fatty acid liberation from VCO hydrolysis at different reaction temperatures.

**Figure 7:**
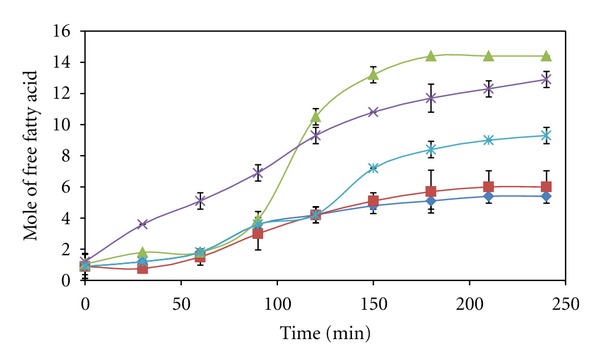
Reaction progress curves of VCO hydrolysis catalyzed by Lipozyme at different reaction temperatures from 30 (♦), 35 (■), 40 (▲), 45 (×) to 50°C (∗).

According to the Arrhenius equation, the reaction rate increases with the increase of temperature. The increase in temperature has accelerated the mobility of substrate and product, thereby increasing the initial reaction rate from 30 to 45°C as presented in Figure 6. Even though 45°C was the temperature that could produce the highest initial reaction rate, it was not the temperature that would produce the highest amount of free fatty acids after 6 hours of reaction (Figure 7). Lipozyme most probably could not stand at 45°C for long time of reaction. After 100 hours of reaction, the rate of VCO hydrolysis at 40°C was increased significantly and overtook the hydrolysis rate at 45°C. Therefore, it produced higher amount of free fatty acids compared to the hydrolysis with the highest initial reaction rate at 45°C. In order to reuse the enzyme for repeated cycles, it is necessary to carry out the reaction at 40°C by compensating the initial reaction rate.

However, the initial reaction rate decreased when the temperature was increased beyond 45°C. This was most likely due to the deactivation of the enzyme. It is known that most proteins tend to denature at temperatures above 50°C [1]. In addition, the presence of deactivated enzyme at the interface would block the active enzymes from penetrating to the interface [1]. 

## 4. Conclusions

VCO hydrolysis catalyzed by immobilized lipase had been investigated by varying the reaction parameters such as VCO concentration, enzyme loading, water, and temperature. The profile of each parameter showed a bell shape curve, where the initial reaction rate was increased up to a critical value and subsequently the hydrolysis rate was decreased due to the unfavorable effects of extreme reaction parameters. Based on the results of initial velocity of reaction, the optimum conditions for the hydrolysis were as follow: VCO concentration at 40% (v/v), enzyme loading at 1% (w/v), water content at 7% (v/v), and reaction temperature at 45°C. In all experiments, it seems that solvent free system was unable to produce compatible results as the solvent-based system. The solvent-free system showed poor performance in terms of the final yield of free fatty acid production as well as the initial reaction rate.
